# Metabolic Engineering of Bacterial Respiration: High vs. Low P/O and the Case of *Zymomonas mobilis*

**DOI:** 10.3389/fbioe.2019.00327

**Published:** 2019-11-12

**Authors:** Uldis Kalnenieks, Elina Balodite, Reinis Rutkis

**Affiliations:** Institute of Microbiology and Biotechnology, University of Latvia, Riga, Latvia

**Keywords:** respiratory chain, metabolic engineering, energy coupling, redox balance, stress resistence, *Zymomonas mobilis*

## Abstract

Respiratory chain plays a pivotal role in the energy and redox balance of aerobic bacteria. By engineering respiration, it is possible to alter the efficiency of energy generation and intracellular redox state, and thus affect the key bioprocess parameters: cell yield, productivity and stress resistance. Here we summarize the current metabolic engineering and synthetic biology approaches to bacterial respiratory metabolism, with a special focus on the respiratory chain of the ethanologenic bacterium *Zymomonas mobilis*. Electron transport in *Z. mobilis* can serve as a model system of bacterial respiration with low oxidative phosphorylation efficiency. Its application for redox balancing and relevance for improvement of stress tolerance are analyzed.

## Introduction

During the last few decades a vast body of evidence on the structure, function and regulation of microbial electron transport chains has accumulated, enabled by the rapid advancement of omics techniques and molecular cloning tools. Research has focussed on deciphering of particular electron pathways in several model microorganisms, establishing their physiological role, and understanding the principles of their regulation. Transcriptional regulation in *Escherichia coli* in response to variation of available electron acceptors perhaps is the best known success story (for reviews, see Spiro and Guest, 1991; Unden and Bongaerts, 1997; Green and Paget, 2004). Here we briefly discuss how this wealth of theoretical knowledge can be used for industrial applications in the present era of metabolic engineering and synthetic biology. Generally speaking, microbial aerobic electron transport chains represent a promising, yet at the same time, challenging target for rational metabolic engineering, since their activities transcend several key cellular processes, like the catabolic ATP yield, maintenance of intracellular redox balance, production of reactive oxygen species (ROS), and protection against oxidative stress. In the present minireview we attempted to systematize the strategies of respiratory chain engineering, based on their impact on the cellular energy and reducing equivalent balance. We did not review the biotechnological applications of bacterial anaerobic respiration, electron transport chains of extremophiles, as well as bioelectrochemistry—interaction of bacterial electron transport with electrodes. Instead of trying to embrace all aspects of microbial electron transport, we here focussed on a few current biotechnology workhorses, showing how the metabolic engineering of their respiration had yielded predictable (or sometimes, not so predictable) effects on their growth and production. Some emphasis was put on low energy-coupling electron transport and the bacterium *Zymomonas mobilis* as a representative of the energetically uncoupled respiration.

## Respiratory Engineering to Improve Fermentative Catabolism

Microbial respiration represents a major sink of reducing equivalents and a source of energy (ATP) for the cellular metabolism. However, if the target compound of a bioprocess is produced by a redox-balanced fermentative pathway, then respiration, by interfering with the cellular redox and energy balance, causes a decrease of product yield, to mention Pasteur effect as a classical example (for a review, see Barnett and Entian, 2005). NADH supply of reactions, producing the desired target compound of fermentative catabolism, often represents a bottleneck (Zhao et al., 2017), especially under aerobic or microaerobic conditions (Bennett and San, 2017). Removing oxygen from the growth medium seems a straightforward approach for turning off the unwanted respiration and fueling more NADH into the fermentative catabolism. However, prolonged maintenance of a strict anoxic condition may be a challenging task. It may complicate, for example, long-term directed evolution experiments, like those aimed at engineering of the *E. coli* fermentative metabolism (Portnoy et al., 2008), where complete absence of oxygen is essential. On the other hand, many yeast species performing ethanol fermentation require limited presence of oxygen (Visser et al., 1990), often within a certain narrow concentration range for optimum ethanol yield (Acevedo et al., 2017). From the technical point of view, keeping low, steady, micro-aerobic oxygen levels in bioreactors (especially for larger volumes) may be difficult, due to the sensitivity of oxygen mass transfer coefficient to the agitation rate, insufficient responsiveness of the dissolved oxygen probe and spatial in homogeneities (Wu et al., 2015a).

Decreasing the activity of electron transport chain itself represents a viable alternative way to downregulate respiration, so that the cells would behave similar to that of anoxic or microaerobic environment even in the presence of oxygen excess (Bennett and San, 2017). Such a metabolic engineering strategy of limiting the use of oxygen by the cell itself would be useful also under some co-cultivation settings, e.g., when an end-product of fermentative catabolism of one microorganism species is used as a substrate for aerobic metabolism of the second, co-cultivated species. Decreasing of respiration may be achieved by respiratory inhibitors, keeping in mind that inhibitor effects may serve as a proof of principle, yet are hardly applicable to industrial bioprocesses. Thus, Acevedo et al. (2017), using rotenone, the inhibitor of the mitochondrial NADH dehydrogenase complex I, demonstrated an increase of aerobic ethanol yield from xylose in the yeast *Scheffersomyces stipitis*. Cyanide at submillimolar concentrations was shown to improve growth and ethanol yield in aerobic culture of ethanologenic bacterium *Zymomonas mobilis* (Kalnenieks et al., 2000).

Not surprisingly, construction and study of respiratory knock-out mutants nowadays has become the dominating approach to the downregulation of microbial respiration, and has brought positive results. In *E. coli*, removal of all its terminal oxidases, followed by adaptive evolution, yielded strains able to produce lactate as a fermentation product from glucose and to undergo mixed-acid fermentation during aerobic growth (Portnoy et al., 2008). For *Corynebacterium glutamicum*, another important biotechnological producer, a respiratory chain mutant lacking both the cytochrome *bd* branch and the cytochrome *bc*_1_-*aa*_3_ branch was constructed (Koch-Koerfges et al., 2013), and its ability of fermentative growth without aerobic respiration was demonstrated. The mutant retained only 2% of the wild type oxygen consumption rate and displayed fermentative catabolism with L-lactate as major and acetate and succinate as minor products. In aerobically grown *Klebsiella pneumoniae* knocking out of its NADH dehydrogenase complex I (*nuo*) improved 2,3-butanediol production (Zhang et al., 2018). In the ethanol-producing, facultatively anaerobic bacterium *Z. mobilis* inactivation of its Type II respiratory NADH dehydrogenase (*ndh*) largely improved growth and ethanol yield under aerobic conditions (Kalnenieks et al., 2008; Hayashi et al., 2011, 2012; see below).

Some metabolic engineering studies have attempted fine-tuning of the respiration rate in *E. coli*. In order to mimic microaerobic metabolism of *E. coli* under condition of excessive aeration, Zhu et al. (2011) manipulated respiration rate of cells by externally adding various amount of coenzyme Q_1_ to a strain lacking the *ubiCA* genes necessary for quinone biosynthesis. They demonstrated that synthesis of fermentation products, like lactate and ethanol, varied inversely with the added amount of Q_1_. In the further study (Wu et al., 2015a), a nearly theoretical yield of lactate production was achieved under fully aerobic conditions by introducing a tunable pathway competing with biosynthesis of the precursors of coenzyme Q_8_. This regulatory strategy was named by authors “metabolic transistor” (Wu et al., 2015b), allowing to control a large flux (electron transport) by a small change in the availability of one of the electron transport components (Q_8_). An approach somewhat similar to that of Zhu et al. (2011) has been applied to *Lactococcus lactis*, in which the activity of respiratory chain naturally depends on externally added hemin, the precursor of the cytochrome heme cofactors (Liu et al., 2017). The authors constructed a multiple dehydrogenase knock-out strain of *L. lactis* with its respiratory chain as the only remaining NADH sink, and showed how the external control of respiration rate by hemin affected its growth and the degree of reduction of the synthesized products (acetoin vs. butanediol).

Bacterial respiratory chains can be engineered to control NADH/NAD^+^ ratio and reducing equivalent fluxes also under anaerobic conditions. For enhancing NADH-dependent fermentative production in some strict anaerobes, the activity of electron transport chain could be used to generate NADH at the expense of other reduced cofactors, e.g., ferredoxin. This approach recently was demonstrated in *Clostridium thermocellum* (Lo et al., 2017), and in the extreme thermophile *Caldicellulosiruptor bescii* (Williams-Rhaesa et al., 2018), in which conversion of pyruvate to acetyl-CoA proceeds largely via pyruvate:ferredoxin oxidoreductase. By overexpressing Rnf, the membrane bound energy-conserving ferredoxin:NAD^+^ oxidoreductase, NADH flux to the NADH-dependent ethanol synthesis could be improved, leading to a substantial increase of ethanol production in both these bacteria, and a concomitant decrease of production of unwanted side-products.

Notably, some recent works on modification of respiratory NADH dehydrogenase activity in aerobic or facultatively anaerobic bacteria reported unexpected, NADH dehydrogenase-dependent metabolic shifts under strictly anaerobic conditions, which at present seem difficult to explain. Under aerobic conditions, manipulating the activity of either the NADH dehydrogenase complex I (*nuo*) or the Type II NADH dehydrogenase (*ndh*) was shown to affect the intracellular NADH/NAD^+^ ratio, and accordingly, the catabolic product spectrum in a fairly predictable manner, as reported for several bacteria, e.g., *Bacillus subtilis* (Gyan et al., 2006), *E. coli* (Liu Q. et al., 2014), *K. pneumoniae* (Zhang et al., 2018), and *Streptococcus agalactiae* (Lencina et al., 2018). Yet, for example in *C. glutamicum*, overexpression of the *ndh* gene under oxygen-deprived conditions also substantially decreased the NADH/NAD^+^ ratio, at the same time increasing the glucose consumption rate (Tsuge et al., 2015). Here “oxygen-deprived conditions” referred to <0.01 ppm oxygen, and importantly, no alternative terminal electron acceptors were present in the medium. Similarly, Hayashi et al. (2015) found a strong dependence of NADH/NAD^+^ ratio on the Ndh activity in complemented *Z. mobilis ndh* mutant strains under strictly anaerobic conditions. No electron acceptors alternative to oxygen are known for this bacterium. Steinsiek et al. (2014) noted that inactivation of the NADH dehydrogenase complex I (*nuo*) in *E. coli* affected not only its aerobic metabolism, but induced significant metabolic response also under anaerobic conditions, and without alternative external electron acceptors—again, conditions where the respiratory chain should not be active. They observed genes coding for TCA cycle enzymes being upregulated, and higher amounts of succinate being synthesized by the mutant. At the same time, they noted a substantial shift of the ArcA phosphorylation pattern in the *nuo* mutant relative to the parent strain. Earlier Perrenoud and Sauer (2005) demonstrated that ArcA-dependent transcriptional regulation controls TCA cycle in *E. coli* not only under (micro)aerobic, but also under fully anaerobic conditions. Yun et al. (2005) reported higher lactate and formate yields in *E. coli ndh* mutant and *ndh nuo* double mutant under strictly anaerobic conditions. Apparently, more research is needed to understand these anaerobic effects and their possible relation to the global regulatory mechanisms linking respiratory chain to the central metabolism. At the same time, it is important to take them into account for practical respiratory engineering.

## Improving Respiratory Energy-coupling

The classical work of Bauchop and Elsden (1960) provided evidence that on average one mole of ATP could fuel synthesis of slightly above 10_gdrywt_ of bacterial biomass. With a few exceptions, this value (Y_ATP_) was found to be roughly similar for different microorganisms (Stouthamer, 1977). The apparent Y_ATP_ is far from being a true “biological constant” for all growth conditions, since it depends on cellular maintenance energy requirements and energy-spilling reactions (Russell and Cook, 1995). Yet, primarily it is the variation of energy generation efficiency, but not that of Y_ATP_, that underlies the observed vast differences of microbial growth yields. Regarding aerobic catabolism, the efficiency of energy generation is determined by the stoichiometry of oxidative phosphorylation (Calhoun et al., 1993). The stoichiometry of oxidative phosphorylation (P/O) is the molar ratio between the generated ATP macroergic phosphate bonds and the oxygen atoms reduced to water by the respiratory chain (Hinkle, 2005). The mechanistic (maximum) P/O value for each particular case can be estimated from the known proton motive stoichiometries of the respiratory complexes and H^+^-ATP synthase, keeping in mind that the real values are lower due to proton leaks and slips in the energy-coupling mechanisms. Typically, the bacterial electron transport chains are branched, and for electrons traveling from NADH via the respiratory complexes to oxygen, their contribution to energy coupling strongly depends on the particular pathway that the electrons take. For example, in *E. coli* electron transport chain (for a review, see Unden and Bongaerts, 1997) the stoichiometry of proton translocation per one transported electron (H^+^/e) may vary between 1 and 4 (accordingly, H^+^/O varies between 2 and 8). Assuming the proton/ATP stoichiometry of the H^+^-ATP synthase close to 3 (Kashket, 1982), the theoretical lower and upper limits for P/O in *E. coli* appear to be 0.67 and 2.67, respectively. In reality, *E. coli* and most other bacteria with branched respiratory chains tend to employ their energetically most efficient and less efficient electron transport branches simultaneously, resulting in a submaximal net energy coupling efficiency, which is hard to measure directly under *in vivo* condition. A metabolic network model with a growth rate-dependent biomass composition, used for simulation of *E. coli* chemostat cultivations on several carbon substrates at different dilution rates, has yielded an estimate of P/O close to 1.5 (Taymaz-Nikerel et al., 2010). Thermodynamic analysis suggests that the submaximal P/O values, typically in the range of 1.0–1.5, indicate a tradeoff between metabolic energy conservation and energy dissipation, ultimately directed toward maximization of the microbial growth rate (Westerhoff et al., 1983; Molenaar et al., 2009; Werner et al., 2010).

**Figure 1:**
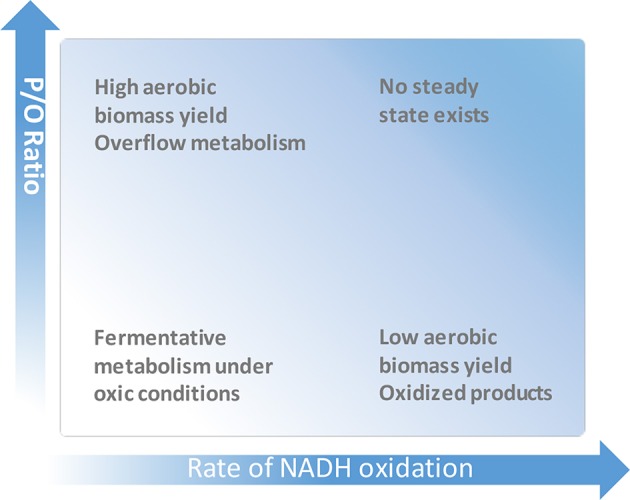
Dependence of microbial growth and production on the energetical efficiency of respiration.

From a practical point of view, increasing of P/O represents a metabolic engineering strategy for improving microbial biomass yield (Figure 1). For increase of P/O, the electron transport branches with low coupling efficiency have to be inactivated, and/or those with high efficiency have to be overexpressed. Biomass yield (and in some cases, e.g., in lactic acid bacteria, also multiple stress tolerance; Zotta et al., 2013) can thus be improved, which is desirable for cost-efficient biomass production of starter cultures, for the net productivity of bioconversions, and for the production of biomass-related compounds such as proteins, storage polymers and vitamins (Minohara et al., 2002; Brooijmans et al., 2007; Pedersen et al., 2012; Richhardt et al., 2013; Liu Q. et al., 2014).

Apart from biomass, high P/O ratio has been shown to push the synthesis of various products via pathways that consume (or at least, do not produce) ATP (de Kok et al., 2012). This takes place because the energetically efficient respiration minimizes the amount of carbon substrate that has to be oxidized to generate ATP for needs of growth and maintenance. Given that the carbon source is not limiting and its uptake is rapid enough, the high P/O may facilitate production of incompletely oxidized compounds, either target products or, sometimes, undesired inhibitory metabolites. This phenomenon is known as “overflow metabolism” (Neijssel and Tempest, 1976; Figure 1). Enhancing product synthesis by elevating P/O has been a strategy of quite a few metabolic engineering projects. In most cases, elevation of P/O is achieved by knocking out the energetically least efficient terminal oxidase (typically cytochrome *bd*), and thus redirecting electron flow to energetically more efficient terminal oxidase(s). Poly-γ-glutamic acid (γ-PGA), a multifunctional biopolymer with various applications, is an example of a product, for which ATP supply is essential for its biosynthesis. By elimination of the cytochrome *bd* branch in *Bacillus licheniformis* respiratory chain, Cai et al. (2018) reached an increase of γ-PGA yield by almost 20%. Efficient supply of ATP is critical also for production of riboflavin. In a fed-batch culture of riboflavin-producing *B. subtilis* a 30% higher riboflavin titer was accumulated by the *cyd* mutant compared to the control strain (Zamboni et al., 2003), apparently because of redirection of the electron flow to the proton-pumping *aa*_3_ terminal oxidase. Similar approach was used for improving of N-acetylglucosamine production in *B. subtilis* (Liu Y. et al., 2014) and lysine yield in *C. glutamicum* (Kabus et al., 2007). Liu Q. et al. (2014) demonstrated that inactivation of the cytochrome *bd-*II terminal oxidase and/or the type II (energy non-coupling) respiratory NADH dehydrogenase results in a several-fold increase of poly(3-hydroxybutyrate) accumulation in an *E. coli* recombinant producer strain.

However, under some natural conditions, and also in numerous bioprocess setups it may be preferable for bacteria to have respiration with low rather than high P/O.

## Increasing Respiration Rate While Decreasing Energy-coupling Efficiency

Low P/O is preferable when oxidation of NAD(P)H is needed for a rapid regeneration of NAD(P)^+^ and/or consumption of oxygen, without producing of extra ATP (Figure 1). Thus, the free-living nitrogen-fixing bacteria *Azotobacter vinelandii* demonstrate the utility of rapid, low-P/O respiration as one of possible means to protect their nitrogenase complex from dioxygen by keeping the intracellular oxygen concentration low (Poole and Hill, 1997). It was shown that the activity of the low energy-coupling cytochrome *bd* terminal oxidase (Kelly et al., 1990) in combination with an energy non-coupling NADH:UQ oxidoreductase (Bertsova et al., 2001) is critical for *A. vinelandii* ability to fix nitrogen while growing under conditions of air saturation. In bioprocesses, energetically uncoupled respiration is useful for avoiding production of unneeded biomass and overflow metabolites, while enabling conversion of reduced substrate into more oxidized product(s). The obligately aerobic alpha-proteobacterium *Gluconbacter oxydans* belonging to the family of acetic acid bacteria is a relevant example. Due to its numerous periplasmic membrane-bound dehydrogenases that incompletely oxidize sugars, sugar alcohols, and other compounds, *G. oxydans* has been used for decades as a biocatalyst for bioconversions (Deppenmeier et al., 2002). It oxidizes a variety of substrates and transfers the electrons via ubiquinone to the quinol oxidases *bo*_3_ and *bd*. Although the energetically more efficient, proton-pumping *bo*_3_ represents its major terminal oxidase activity (Richhardt et al., 2013), due to some reason the proton pumping stoichiometry of the *G. oxydans* respiratory chain is more than two-fold lower than that of aerobically cultivated *E. coli* bearing analogous major terminal oxidase.

Seeking to relate the overflow metabolism phenomenon in the model microorganisms *E. coli* and *S. cerevisiae* to their respiratory capacity, Vemuri et al. (2006a, 2007) demonstrated that overexpression of plant non-coupling alternative terminal oxidases, and/or the energy non-coupling, soluble water-forming NADH oxidase from *Streptococcus mutans* reduces the overflow metabolism, decreases the biomass yield, and shifts catabolism toward more oxidized products. In *E. coli* overexpression of streptococcal NADH oxidase was shown to raise the TCA cycle flux and to elevate the NADPH/NADP^+^ ratio (Holm et al., 2010). In combination with the deletion of *arcA* gene it dramatically reduced acetate accumulation and hence improved capacity of recombinant *E. coli* to overexpress heterologous proteins (Vemuri et al., 2006b). Expression of the soluble water-forming NADH oxidases (*nox*) of *Streptococci* or *Lactococci*, which do not contribute to energy coupling, has been widely applied in metabolic engineering projects for enhancing respiration, lowering its energy-coupling efficiency, and decreasing the intracellular NADH/NAD^+^ ratio. Overexpression of *noxE* from *L. lactis* in *Saccharomyces cerevisiae* has been shown to shift catabolism toward more oxidized products, like acetaldehyde, acetate, and acetoin (Heux et al., 2006). NoxE overexpression prevents accumulation of reduced byproducts, such as glycerol and xylitol, yet improves ethanol yield during xylose fermentation by engineered *S. cerevisiae* (Zhang et al., 2017), as well as improves oxidative and osmotic stress tolerance of *S. cerevisiae* aerobic batch culture on glucose (Shi et al., 2016). The same oxidase has been overexpressed in *E. coli* (Balagurunathan et al., 2018) and in *L. lactis* (Bongers et al., 2005) to increase acetaldehyde yield at the expense of more reduced products of sugar catabolism. Bacterial soluble NADH oxidases are recently used also for *in vitro* metabolic pathways, reconstituted with isolated enzymes, as the means for recycling NADH (Nowak et al., 2015; Taniguchi et al., 2017).

In a broader sense, the metabolic engineering strategy for uncoupling of energy metabolism is not confined merely to manipulating the electron transport modules. A decrease of the energy-coupling efficiency can be achieved also by targeting the proton-dependent ATPase in order to disrupt oxidative phosphorylation, or to stimulate ATP hydrolysis. Following this approach, one can achieve energetical uncoupling in combination with overflow metabolism. Indeed, several studies have reported a decrease of biomass yield, growth rate, and intracellular ATP/ADP ratio, an increase of glycolytic and respiratory rates, and a rise of acetate production (overflow) in ATPase-deficient *E. coli* mutants (Jensen and Michelsen, 1992; Noda et al., 2006). Noda et al. (2006) observed activation of the low energy-coupling modules in the electron transport chain of such mutant: the type II NADH dehydrogenase and cytochrome *bd* terminal oxidase were upregulated. The reason for that might be the need for rapid regeneration of NAD^+^ to keep the rate of glycolysis high, while at the same time investing less into proton motive force generation, since it no more could drive ATP synthesis. A similar shift in respiratory chain composition and an onset of acetate overflow were reported by Schuhmacher et al. (2014) in a wild type *E. coli*, grown under phosphate limiting condition. Notably, these physiological effects of phosphate limitation, resembling the ATPase knock-out phenotype, correspond to earlier *in vitro* findings with membrane preparations (Turina et al., 2004; D'Alessandro et al., 2008); and reconstituted liposome systems (D'Alessandro et al., 2011), indicating that H^+^-ATPases of *E. coli* and *Rhodobacter capsulatus* at low phosphate and ADP concentrations decouple ATP hydrolysis from transmembrane proton translocation. Causey et al. (2003) employed the acetate overflow effect for metabolic engineering of an acetate-producing *E. coli*. By inactivating the H^+^-ATP synthase along with disruption of the TCA cycle and elimination of native fermentative pathways, they obtained a strain, which synthesized acetate as its major product, reaching 86% of the theoretical maximum yield. Apart from *E. coli*, a somewhat similar response to inactivation of ATP synthase has been documented in other biotechnologically important bacteria, like *C. glutamicum* and *Bacillus subtillis* (Santana et al., 1994; Koch-Koerfges et al., 2012).

Alternatively, instead of disrupting oxidative phosphorylation, one can decrease the energetic efficiency of metabolism just by stimulating futile hydrolysis of ATP. In their pioneering work (Koebmann et al., 2002a,b) overexpressed the F_1_ part of H^+^-ATPase, which could be used as an universal module for metabolic engineering of intracellular ATP hydrolysis without interfering with any particular metabolic reaction. In *E. coli* and *L. lactis* overexpression of F_1_ led to a decrease of growth rate and intracellular energy level, and (depending on the strain and culture growth phase) also stimulated glycolytic flux to a various degree, decoupling it from anabolism. Using this approach Liu et al. (2016) demonstrated that futile hydrolysis of ATP could enhance product synthesis via pathways that generate ATP. By overexpressing of F_1_ in *L. lactis* strain engineered for acetoin production they managed to improve the product yield and productivity at the expense of biomass formation. As noted by Holm et al. (2010), the metabolic effects of overexpression of the F_1_ part of H^+^-ATPase and those of the soluble water-forming NADH oxidase had much in common. In both cases for *E. coli* the result was an elevated glucose consumption and reduced growth rate and biomass yield, although the acetate overflow was seen only with F_1_, but not with Nox.

Finally, since majority of bacterial electron transport chains are branched (for a review, see Poole and Cook, 2000), in principle it should be possible to decrease P/O also by inactivation of the energetically efficient electron transport branch(es), while leaving the less efficient, low-coupling ones active. However, in several cases inactivation of the energetically efficient routes has resulted in a drop of the bulk activity of mutant respiratory chain, actually causing poor growth and an elevation of overflow metabolism. Reported examples are the *E. coli* strains with linear electron transport chains (Steinsiek et al., 2014), or *aa*_3_ knock-out mutant of *B. subtilis* (Zamboni and Sauer, 2003), bearing the least energetically efficient electron transport components, yet accumulating more acetate than the respective wild types. Hence, turning a natural bacterial respiratory chain with an average P/O value into a low-P/O and high-rate one often implies complex genetic modifications and fine-tuning, including knocking out the high-coupling modules and concerted overexpression of low-coupling respiratory chain constituents. Obviously, the best alternative to such complex genetic engineering might be employing bacterial respiratory chains with a naturally low P/O, whenever possible. *L. lactis* is one of such bacteria, bearing a low energy-coupling, inducible respiratory chain, suitable for oxidizing surplus NADH, while little affecting cellular ATP production (Liu et al., 2017). Notably, although NoxE from this bacterium has been expressed in several microorganisms to elevate their respiration rate (see above), its own inducible respiratory chain proved to be more efficient than NoxE for regeneration of its own intracellular NAD^+^ pool and for its oxidative stress protection. The ethanol-producing alpha-proteobacterium *Z. mobilis* is another prominent example of aerobic metabolism with a naturally uncoupled respiration.

## *Zymomonas mobilis*: an Example of Uncoupled Energy Metabolism

*Zymomonas mobilis* is a facultatively anaerobic bacterium with a very rapid and efficient homoethanol fermentation pathway (Rogers et al., 1982), involving the Entner-Doudoroff (E-D) glycolysis, pyruvate decarboxylase, and two alcohol dehydrogenase isoenzymes (Sprenger, 1996). Under anaerobic or microaerobic conditions up to 98% of substrate carbon is incorporated in ethanol. Metabolic engineering of *Z. mobilis* has largely focussed on biofuel production from variety of renewable substrates, as well as on increasing its stress-resistance (Zhang et al., 1995; Mohagheghi et al., 2015; Yang et al., 2016; Charoensuk et al., 2017; for recent reviews, see Wang et al., 2018; Xia et al., 2019). Fermentative catabolism in *Z. mobilis* is loosely linked to its anabolism and growth, and proceeds with a high rate, both during the exponential growth phase, or under non- or slowly-growing conditions (Belaich and Senez, 1965). With respect to its metabolism, *Z. mobilis* somewhat resembles lactic acid bacteria (Sauer et al., 2017), also having high carbon substrate uptake rates with low biomass formation in combination with simple central metabolic pathways, leading to a limited number of metabolites. However, the aerobic energy metabolism in *Z. mobilis* seems to stand further away from the “bacterial mainstream,” than that of lactic acid bacteria.

**Figure 2:**
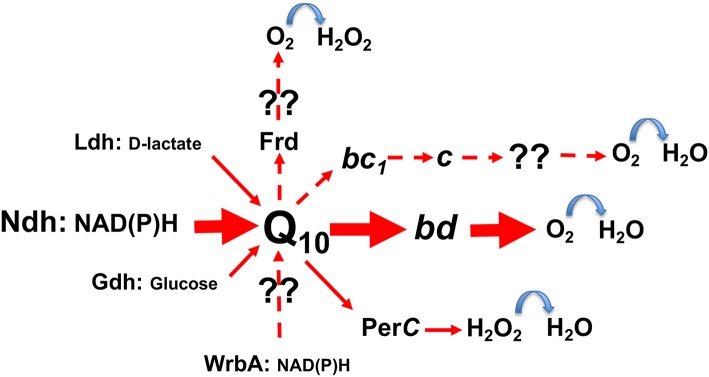
The scheme of electron transport pathways in *Zymomonas mobilis* respiratory chain. Hypothetical pathways are shown with dashed arrows. Frd, putative fumarate reductase; WrbA, putative type IV NAD(P)H:quinone oxidoreductase; for other abbreviations see text.

In contrast to the inducible electron transport of lactic acid bacteria, which requires hemin to be present in growth media (Brooijmans et al., 2007; Koebmann et al., 2008), *Z. mobilis* bears a constitutive respiratory chain. It consists of the Type II NADH dehydrogenase (*ndh*), coenzyme Q_10_, and the cytochrome *bd* terminal oxidase (*cydAB*) as the established major electron carriers (Figure 2), together with respiratory FAD-dependent D-lactate dehydrogenase (Ldh), PQQ-dependent glucose dehydrogenase (Gdh), a putative cytochrome *c* peroxidase (PerC), and some other minor or still unidentified constituents (Strohdeicher et al., 1990; Kalnenieks et al., 1998; Sootsuwan et al., 2008). The cytochrome *bd* seems to be the only terminal oxidase, which is encoded in the *Z. mobilis* genome (Seo et al., 2005; Yang et al., 2009), and has been detected spetroscopically (Kalnenieks et al., 1998; Sootsuwan et al., 2008). *Z. mobilis*, like *G. oxydans* (Hanke et al., 2012) and some *Acetobacter* species, possesses a “dead-end” cytochrome *bc*_1_ branch, apparently lacking a terminal cytochrome *c* oxidase, hence its function in electron transport remains obscure. The cytochrome *c* peroxidase seems more likely to function as a quinol peroxidase (Balodite et al., 2014), than to terminate the *bc*_1_ electron transport branch, as suggested by Charoensuk et al. (2011). Nevertheless, *Z. mobilis* mutant strain with inactivated cytochrome *b* subunit of the *bc*_1_ complex shows a distinct respiratory phenotype (Strazdina et al., 2012), best seen in mutant membrane preparations as an increase of antimycin-resistance of oxygen consumption and alteration of cytochrome *bd* reduction kinetics with NADH. In *G. oxydans* its *bc*_1_-deficient strain shows a growth defect under acidic conditions, while in the wild type the *qcrABC* genes are upregulated under oxygen limitation (Hanke et al., 2012). Further research might reveal novel roles for the *bc*_1_ complex in these alpha-proteobacteria and perhaps, put forward new ideas for their respiratory metabolic engineering.

For some reasons, the energetic efficiency of *Zymomonas* respiration in aerated culture is significantly lower than reported for other bacteria, e.g., *L. lactis*, with a similar dominating electron transport pathway from Ndh to CydAB (Brooijmans et al., 2007; Rutkis et al., 2014, 2016), which makes it quite unique among the producer microorganisms. Unlike such prominent facultatively anaerobic workhorses of biotechnology as *E. coli* and *B. subtilis*, aeration does not improve *Z. mobilis* biomass yield (Belaich and Senez, 1965; for a review, see Kalnenieks, 2006). Study of respiratory knock-out mutants (Strazdina et al., 2012; Rutkis et al., 2014) did not indicate presence of any putative low energy-coupling electron transport branch, to which one might ascribe the overall low energetic efficiency. Oxidative phosphorylation has been reported in non-growing cells and cell membrane vesicles, and cells are capable of ATP synthesis with artificially induced proton gradient (Kalnenieks et al., 1993). Yet, the proton-dependent ATPase in *Z. mobilis* under aerobic growth conditions operates in the direction of ATP hydrolysis (Rutkis et al., 2014). This is possibly because of some intrinsic deficiency of proton motive force maintenance, or due to an unidentified proton leakage pathway in growing cells. Being an active sink of reducing equivalents, respiration lowers the ethanol yield, since it withdraws NADH from the alcohol dehydrogenase reaction. Hence, respiration causes accumulation of acetaldehyde, the inhibitory metabolic precursor of ethanol, which contributes to the poor aerobic growth of this bacterium, at the same time being a valuable target product of *Z. mobilis* aerobic bioprocess (Wecker and Zall, 1987). From the practical point of view, respiratory chain of *Z. mobilis* is well-suited for oxidation of NADH (and NADPH, since its Ndh has a good affinity for both cofactors) without stimulation of biomass growth. Aerobic acetaldehyde production by *Z. mobilis* so far is the best reported application of its respiratory potential. Recently, acetaldehyde-producing strains were developed, that reach 70% of the theoretical maximum acetaldehyde yield (Kalnenieks et al., 2019), as well as strains with the acetaldehyde-generating pyruvate decarboxylase reaction relocated to periplasm, in order to mitigate toxic effect of acetaldehyde on cell interior (Balodite et al., 2019).

It is not obvious, what are the physiological function(s) of *Z. mobilis* respiration in nature. Possibly, the respiratory chain might be accelerating glucose consumption by lowering the intracellular NADH/NAD^+^ ratio (Rutkis et al., 2016), like it has been shown previously for *C. glutamicum* (Tsuge et al., 2015). Rutkis et al. demonstrated that an active respiratory chain is advantageous for reaching higher initial glucose uptake rate in non-growing cells, thus improving their survival, when glucose is available in small amounts and for restricted periods of time (most likely, that is what happens during the natural life cycle of this bacterium).

Respiratory chain apparently plays some role in the protection of *Z. mobilis* against oxic, high temperature, inhibitor and salt stresses. Cytochrome *c* peroxidase (*perC*) knock-out mutant of the thremotolerant strain TISTR 548 shows impaired growth at elevated temperature (37 and 39°C) (Charoensuk et al., 2011). Hypersensitivity to hydrogen peroxide (although, not as pronounced as in catalase-deficient strain) is another phenotypic trait of *Z. mobilis perC* mutants (Charoensuk et al., 2011; Balodite et al., 2014). In their recent work with TISTR 548, Charoensuk et al. (2017) presented results of transposon mutagenesis, followed by the mutant screening for the loss of thermotolerance. They identified 26 knock-out mutations, deleterious for thermotolerance, most of which were related to biosynthesis and maintenance of cell membranes and envelope. Yet, among the mutated genes there was the flavoprotein WrbA, a new type (type IV) of NAD(P)H:quinone oxidoreductase (Patridge and Ferry, 2006), thought to function as a protector against oxidative stress. Martien et al. (2019) noted upregulation of all respiratory chain components following exposure of a strictly anaerobic culture of *Z.mobilis* Zm4 to vigorous aeration, and suggested that the activity of PerC and cytochrome *bd* limits the toxic effects of oxygen. Interestingly, Ndh activity was shown to confer advantage for *Z. mobilis* aerobic growth and flocculation on minimal media (Jones-Burrage et al., 2019). Upregulation of *ndh* and some other oxidoreductase genes were observed under lignocellulose inhibitor stress (Chang et al., 2018). Hayashi et al. (2015) demonstrated positive impact of Ndh activity on *Z. mobilis* salt stress resistance. Likewise, activity of respiratory chain is positively correlated with acetic acid tolerance in *Acetobacter pasteurianus* (Wu et al., 2017), with multiple stress tolerance of *Lactobacillus plantarum* (Zotta et al., 2013), and with resistance of *E. coli* to critically high growth temperatures (Murata et al., 2018). Details of the mechanisms behind these general stress-protective effects, however, largely remain obscure and require further study.

Paradoxically, turning off *Z. mobilis* respiration also appears to be beneficial for its aerobic growth. The *Z. mobilis ndh* knock-out mutant shows improved aerobic growth and ethanol synthesis on rich growth medium (Kalnenieks et al., 2008; Hayashi et al., 2011, 2012), which partly can be explained by the decrease of acetaldehyde accumulation. At the same time, inactivation of the *ndh* gene elicits an adaptive response at the level of the electron transport chain and several oxidative stress protection systems (Strazdina et al., 2012). In particular, the mutant bears higher activity of respiratory D-lactate dehydrogenase (Ldh). Hayashi et al. (2011, 2012) analyzed a set of spontaneous *Z. mobilis* respiratory mutants with Ndh-deficiency, and observed their improved aerobic growth and ethanol synthesis at elevated temperature (39°C). Recently Strazdina et al. (2018) demonstrated that the elevated Ldh activity of the *ndh* strain is crucial for its temperature-resistance. Inactivation of *ldh* against the Ndh-deficient background renders the non-respiring double mutant more temperature sensitive than the *ndh* strain, bringing it back to the level of the wild type. The protective function of the respiratory Ldh at elevated temperature needs further examination, in order to establish what specific electron pathway(s) starting from Ldh might be involved in *Z. mobilis* thermotolerance.

## Conclusions and Outlook

Engineering of respiratory chain represents a potent lever to modify metabolism of biotechnological producer microorganisms. Rational metabolic engineering of culture growth and product synthesis, based on targeted modification of bacterial respiratory chain branches has been proven successful in numerous cases. At the same time, engineering of electron pathways can elicit complex responses, particularly concerning physiology of stress resistance, which may be difficult to explain within the present picture of electron transport. Modification of respiratory chain components can cause unpredicted effects on growth, catabolism and intracellular redox balance. Some effects, observed under strictly anaerobic conditions, possibly indicate gaps in our understanding of anaerobic electron transport pathways. Apparently, in order to uncover the full potential of respiratory metabolic engineering, more basic research on the structure and regulatory interactions between electron transport and the rest of microbial metabolism is needed. For broadening our knowledge of the alternative physiological roles of respiration, bacteria like *Z. mobilis*, which do not use their respiration for energy generation, might be helpful as experimental model systems.

## Author Contributions

All authors listed have made a substantial, direct and intellectual contribution to the work, and approved it for publication.

### Conflict of Interest

The authors declare that the research was conducted in the absence of any commercial or financial relationships that could be construed as a potential conflict of interest.
